# Emerging functional principles of tRNA-derived small RNAs and other regulatory small RNAs

**DOI:** 10.1016/j.jbc.2023.105225

**Published:** 2023-09-09

**Authors:** Qi Chen, Tong Zhou

**Affiliations:** 1 Molecular Medicine Program, University of Utah School of Medicine, Salt Lake City, Utah, USA; 2 Division of Urology, Department of Surgery, University of Utah School of Medicine, Salt Lake City, Utah, USA; 3 Department of Human Genetics, University of Utah School of Medicine, Salt Lake City, Utah, USA; 4 Department of Physiology and Cell Biology, University of Nevada, Reno School of Medicine, Reno, Nevada, USA

**Keywords:** tRNA, rRNA, snRNA, snoRNA, Y RNA, vault RNA, PANDORA-seq, small RNA, RNA modification, RNA structure, AGO, aptamer

## Abstract

Recent advancements in small RNA sequencing have unveiled a previously hidden world of regulatory small noncoding RNAs (sncRNAs) that extend beyond the well-studied small interfering RNAs, microRNAs, and piwi-interacting RNAs. This exploration, starting with tRNA-derived small RNAs, has led to the discovery of a diverse universe of sncRNAs derived from various longer structured RNAs such as rRNAs, small nucleolar RNAs, small nuclear RNAs, Y RNAs, and vault RNAs, with exciting uncharted functional possibilities. In this perspective, we discuss the emerging functional principles of sncRNAs beyond the well-known RNAi-like mechanisms, focusing on those that operate independent of linear sequence complementarity but rather function in an aptamer-like fashion. Aptamers use 3D structure for specific interactions with ligands and are modulated by RNA modifications and subcellular environments. Given that aptamer-like sncRNA functions are widespread and present in species lacking RNAi, they may represent an ancient functional principle that predates RNAi. We propose a rethinking of the origin of RNAi and its relationship with these aptamer-like functions in sncRNAs and how these complementary mechanisms shape biological processes. Lastly, the aptamer-like function of sncRNAs highlights the need for caution in using small RNA mimics in research and therapeutics, as their specificity is not restricted solely to linear sequence.

The discovery of regulatory small noncoding RNAs (sncRNAs) has transformed our understanding of gene regulation (1). The most extensively studied sncRNAs, such as small interfering RNAs (siRNAs), microRNAs (miRNAs), and piwi-interacting RNA (2, 3, 4), mainly function through base-pairing with RNA and/or DNA targets to exert RNA-silencing effects in a process called RNAi. In this process, sncRNAs are loaded into a member of the Argonaute family protein (*e.g.*, AGO, PIWI) to recognize complementary sequences in target RNAs (2). This RNAi-based function mode is highly reprogrammable, regulating both endogenous and invasive genes, making it a powerful tool for research and therapeutics. The remarkable advances and interest in RNAi have also triggered the exploration of a wider range of small RNAs, as well as alternative, ancient, and evolutionarily conserved functional principles of sncRNAs. For example, tRNA-derived small RNAs (tsRNAs (5), also known as tRFs (6) or tDRs (7)) have been reported to regulate protein translation and rRNA processing in unicellular species and prokaryotes (8, 9, 10, 11), where essential RNAi components (*e.g.*, Dicer and Ago) are absent, suggesting the presence of alternative functional modes even before the emergence of RNAi.

Recent advances in small RNA sequencing methods have also prompted the consideration of RNAi-independent sncRNA functional modes by revealing a surprising new sncRNA landscape in mammalian tissues and cells, where miRNAs were once considered the dominant sncRNA regulators (1). These new methods have overcome the sequencing bias in traditional methods caused by specific RNA modifications and termini (12, 13), revealing a diverse range of sncRNAs derived from various longer parental RNAs (*e.g.*, tRNAs (5, 14), rRNAs (15, 16), small nucleolar RNAs (snoRNAs) (17, 18), small nuclear RNAs (snRNAs) (13, 19), Y RNAs (20, 21), vault RNAs (22, 23, 24, 25), and mRNAs (26, 27)) (Fig. 1). Moreover, these methods show that compared to the total sncRNA population, miRNAs are in fact a minority in many tissue and cell types in terms of both expression abundance and sequence diversity (12, 13) (Fig. 2).

**Figure 1:**
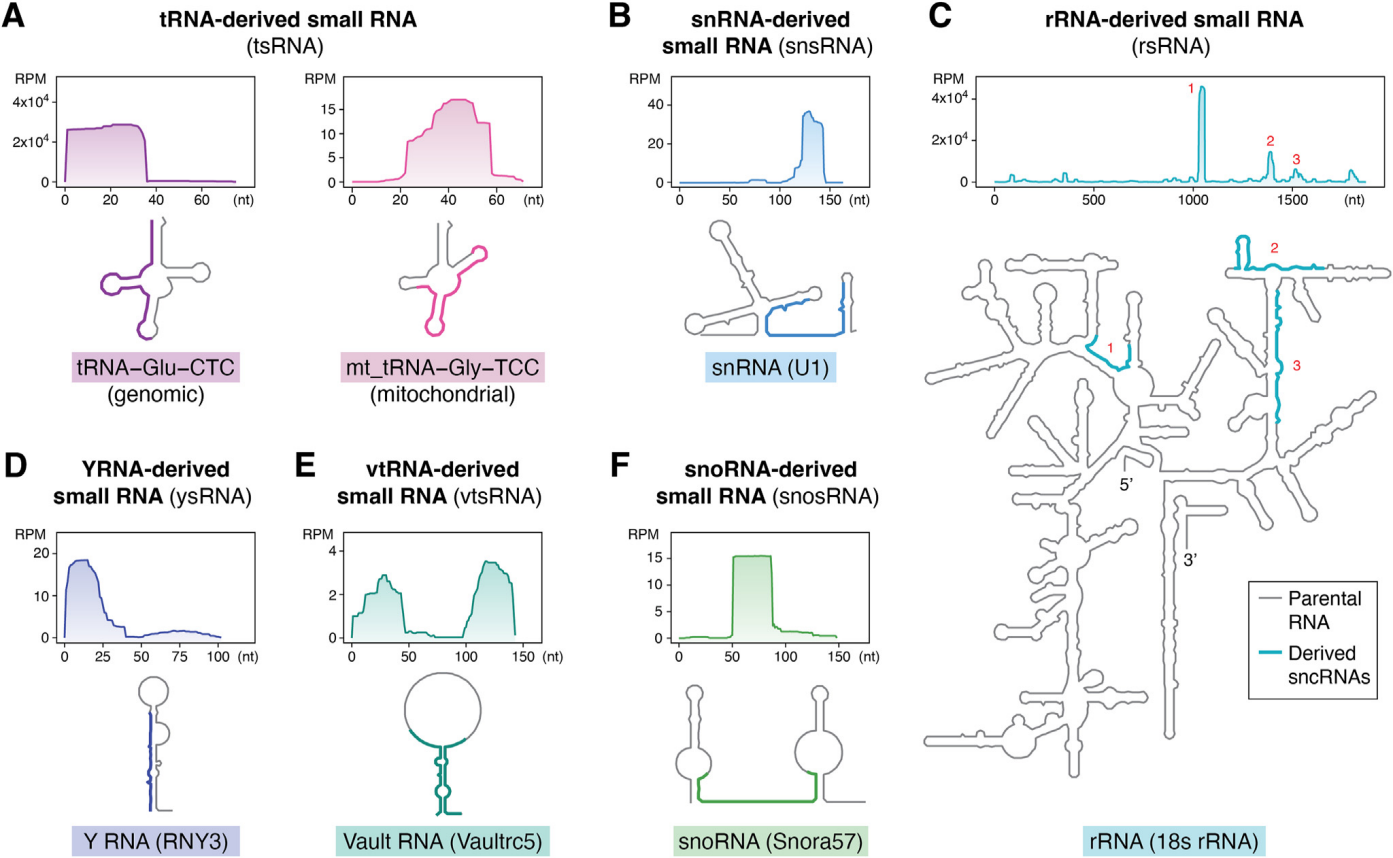
**Schematics of different sncRNAs derived from longer parental RNAs Ref.** (1)**.***A*, tRNA-derived small RNA (tsRNA); (*B*) snRNA-derived small RNA (snsRNA); (*C*) rRNA-derived small RNA (rsRNA); (*D*) Y RNA–derived small RNA (ysRNA); (*E*) vault RNA–derived small RNA (vtsRNA); (*F*) snoRNA-derived small RNA (snosRNA). The *upper section* of each panel illustrates the mapping data of sncRNAs from their parental RNAs, with numbers in the *y*-axis indicating reads per million (RPM). The *lower section* of each panel presents the predicted secondary structure of each parental RNA (depicted in *gray*) according to RNAcentral (https://rnacentral.org). The regions from which sncRNAs are derived are *highlighted* in color, corresponding to the peaks in the *upper section* of the panel. sncRNA, small noncoding RNA.

**Figure 2:**
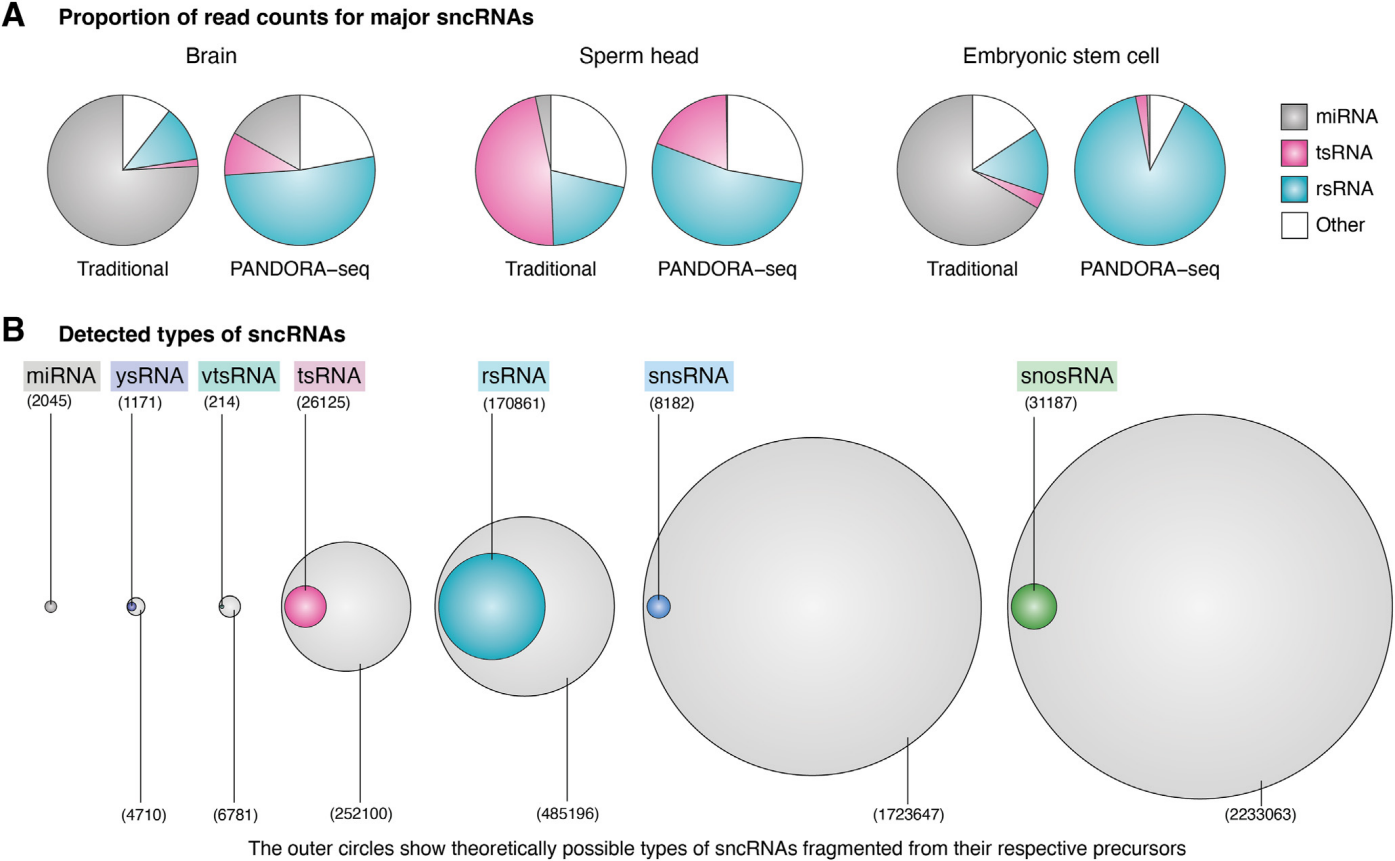
**miRNAs represent a minority of the total sncRNA repertoire.***A*, quantity of different sncRNAs present across various mouse tissues and cells. Data are derived from Ref. (12). *B*, sequence diversity of different sncRNAs in the mouse, including the cumulative sum of mouse miRNA types sourced from miRbase. The types of other detected sncRNAs are compiled from pooled data of tissues and cells provided in Ref. (12). The sncRNAs depicted in the *inner circle* represent detected sncRNAs that appear in at least two independent sequencing libraries. The *outer circles* represent the theoretical possibilities for sncRNA generation, based on potential cleavage at any position of the RNAs (*i.e.*, Y RNAs, vtRNAs, tRNAs, rRNAs, snRNAs, and snoRNAs) to produce sncRNA products of 15 to 44 nucleotides in length. sncRNA, small noncoding RNA; snoRNA, small nucleolar RNA; snRNA, small nuclear RNA; vtRNA, vault RNA.

This expanded sncRNA universe raises new questions about their function, as only a fraction of these newly discovered sncRNAs can bind to AGO and exert RNAi-like functions (1, 5, 15, 28). Factors such as sncRNA length, termini, modifications, and the sncRNA:AGO ratio may account for some sncRNAs not exhibiting RNAi-like functions. In the absence of RNAi, sncRNAs could function through linear base pairing without AGO, or they could act beyond linear base-pairing, adopting an aptamer-like manner based on their 3D structures. This abundant pool of sncRNAs presents both opportunities and challenges for exploring their roles in biology and disease. In this perspective, we aim to consolidate findings from previous reports into new functional principles. We will briefly summarize the progress made in the studies of tsRNAs and other noncanonical sncRNAs and highlight emerging RNAi-independent functions from an aptamer-centric view.

## tsRNA momentum

The journey to recognize the central role of noncoding RNAs in evolution and biology has been long and winding (29). Despite the fact that noncoding sequences are abundant and outnumber protein-coding genes, they were once viewed as insignificant or “junk”. Such biased views may have stemmed from the early ingrained notion that biological functions were primarily carried out by proteins (29). Although we have now moved past the era of considering noncoding RNAs as mere “junk” (30), similar biases may persist in new forms. For example, when the powerful mode of RNAi was discovered along with miRNAs, an emphasis on their biology and utilities, albeit well deserved, led to minimal consideration of other types of sncRNAs with potential alternative modes of action. Yet, sporadic data began to suggest new possibilities, igniting the emerging field of tsRNA research and paving the way for a deeper understanding of the diverse sncRNA landscape (1, 5, 6).

Initial studies on tsRNAs were often accidental, either as a byproduct discovered during analyses of sncRNA sequencing datasets or Northern blot analyses of tRNAs (9, 31, 32, 33, 34, 35, 36). Notably, tRNA fragmentation under various stress conditions has been found in multiple species (9, 10, 31, 32, 34, 36, 37, 38, 39, 40, 41, 42), mediated by various enzymes in different systems as summarized in previous reviews (5, 6). These enzymes encompass a variety of RNases, such as RNase A, T2, Z, and L family members, in addition to Dicer. Moreover, RNA-binding proteins (RBPs), such as the Lupus autoantigen La, an RNA chaperone, can affect the folding of pre-tRNAs and thus the cleavage site for tsRNA biogenesis (43). The absence of La can lead to alternative folding of pre-tRNA and thus generate different set of tsRNAs with different functions. RNA modification enzymes that introduce diverse tRNA modifications can also influence RNase cleavage (5, 6). For example, in mammalian cells, certain tsRNAs are generated by angiogenin (ANG) cleavage of tRNAs at the anticodon loop under stress conditions, whereas RNA modification enzymes such as DNMT2 and NSUN2 mediate the addition of m^5^C modifications within or around the anticodon sequence (44, 45, 46, 47). The presence of m^5^C at these positions increases tRNA stability, while the absence facilitates ANG-mediated tRNA fragmentation (48) and that tsRNAs might be primarily derived from hypomodified tRNAs (49, 50).

In addition to stress conditions, tsRNAs have also been found in various physiological conditions, particularly with high abundance in mammalian sperm (51, 52, 53), serum (54, 55), various body fluids (56), and extracellular vesicles (57, 58, 59, 60, 61). This observation suggests the potential of tsRNAs for a wider scope of functions in addition to stress responses, both inside cells and in cell–cell communication. Because tsRNAs widely exist in blood and other body fluids, many clinical studies have recently harnessed tsRNAs as biomarkers for a range of clinical diseases and conditions (21, 62, 63, 64, 65, 66, 67, 68). In fact, tsRNAs have been found to play important roles in a wide and expanding range of biological and disease conditions including regulating viral infection (69, 70), cancer progression (71, 72, 73), stem cell differentiation (12, 47, 74, 75), epigenetic inheritance (46, 52, 53, 76, 77, 78, 79), and symbiosis (80, 81).

Since tRNAs are ancient and evolutionarily conserved across all domains of life, studies of tsRNAs also extend to unicellular species including prokaryotes and yeast, where RNAi is absent (8, 9, 10, 11). In fact, these studies have also inspired the exploration of tsRNA biogenesis independent of Dicer and new functional modes beyond posttranscriptional silencing effect in an RNAi-like fashion by loading into AGO proteins (18, 33, 81, 82, 83, 84). For example, tsRNAs have been found to inhibit protein translation without complementary target sites in the mRNA (85). Interestingly, it has been noted that the positions from which tsRNAs are cleaved from tRNAs or pre-tRNAs closely determine their functions (43, 86, 87), such as their ability to load into AGO or bind to specific proteins.

Moreover, tsRNAs derived from similar tRNA positions (3′ or 5′ terminus) can have different modes of action depending on their length. For example, synthetic 3′tsRNA-Lys of differing lengths (18 *versus* 22 nt) have distinct roles in inhibiting retrotransposition of endogenous retroviruses (ERVs) in cell culture (88). The 3′tsRNA-Lys-22 nt induces posttranscriptional silencing of the ERV’s protein-coding mRNA in a miRNA-like fashion, whereas the 3′tsRNA-Lys-18 nt does not trigger RNAi-like mRNA degradation or translational inhibition. Instead, it inhibits the RT of ERV by binding to the primer-binding sequence, leading to a block in RT and impeded ERV complementary-DNA (cDNA) synthesis. This diversity of function could be attributed to the stringent loading length requirements of specific AGO proteins (89, 90, 91); and in this case, the shorter tsRNA (18 nt) that cannot be loaded into AGO can still function through sequence complementarity.

In another example, synthetic 5′tsRNA-ala ranging in lengths of 24 to 31 nt can induce translational inhibition by binding to (and replacing) translational initiation factors, while a shorter version (21 nt) fails to exert this function (92), likely due to the absence of the necessary binding motif or RNA structural information for effective binding to these factors. Moreover, 5′tsRNA-Gly and 5′tsRNA-Glu derived from the cell culture medium exhibit resilience to degradation by RNase A, which cleaves single-stranded RNAs (ssRNAs). However, they can be effectively digested by RNase V1, an enzyme that targets double-stranded RNAs (dsRNAs) (93). This finding suggests that at least some tsRNAs may not exist in a linear form but rather contain double-stranded regions, which could be critical for their function.

## Method revolution unveils the hidden majority

Apart from tsRNAs, new types of noncanonical sncRNAs have also been continually discovered. Starting from late 2000s to the early 2010s, various types of small RNAs were identified as originating from the fragmentation of longer structured RNAs, including rRNAs (15, 16, 94), snoRNAs (17, 18), snRNAs (13, 19), Y RNAs (20, 21), vault RNAs (22, 23, 24, 25, 95), and mRNAs (26, 27), across a range of species and tissue/cell types. These noncanonical sncRNAs were initially reported sporadically through bioinformatic analyses of datasets primarily generated to identify siRNA/miRNA/piwi-interacting RNAs, and their existence was not widely recognized in part due to their relatively low abundance when using traditional small RNA sequencing methods. Yet, recent methodological advances (1) driven by a deeper understanding of sncRNA modifications have led to a more comprehensive view of the sncRNA landscape.

A unique aspect of tsRNAs is their RNA modifications, which were not extensively studied in the early days of discovery due to technical challenges. However, the growing field of tsRNA research has inevitably led to a greater focus on these RNA modifications, which are inherited from their parental tRNAs—the largest source of cellular RNA modifications with over 150 distinct types (96, 97, 98). It was not only revealed that RNA modifications affect the biogenesis of tsRNAs by influencing RNase cleavage (summarized previously (5, 6)) but that they affect tsRNA functions (46, 75, 99). It was also found that some tRNA/tsRNA modifications, including m^1^A, m^1^G, m^3^C, and m^2^_2_G, can block the RT process during the generation of cDNA libraries for sequencing (100, 101). In such cases, sncRNAs carrying these modifications will not be detected by sequencing. In addition, tsRNAs are generated by tRNA cleavage with different RNases, many of which produce tsRNA termini (*e.g.*, 5′-hydroxyl and 2′,3′-cyclic phosphate) that are distinct from those of siRNAs/miRNA (5′-phosphate and 3′-hydroxyl, which are generated by Dicer cleavage) (73). These special tsRNA termini prevent the adaptor ligation process that is necessary for cDNA library generation and thus the tsRNAs with these termini will not be detected. Consequently, these internal and terminal RNA modifications introduce substantial biases in the traditional small RNA sequencing result, meaning that those sncRNAs carrying specific modifications or exhibiting unique termini cannot be systematically identified or accurately quantified.

To overcome these problems, new methods such as PANDORA-seq (12) and others (13, 73, 100, 101, 102, 103, 104, 105, 106) (summarized in review (1)) have recently been developed to overcome adaptor ligation–blocking and RT-blocking RNA modifications. These methods use enzymatic treatments (*e.g.*, sequential use of T4PNK and AlkB) to remove RNA modifications and convert termini and/or using special RT enzymes (*e.g.*, TGIRT, BoMoC, and MarathonRT) that can switch template during RT and read through the modifications. These advances have increased the detection of tsRNAs and substantially updated the sncRNA landscape of other noncanonical sncRNAs, especially rRNA-derived small RNAs (rsRNAs), which are found to be dominant in many cell/tissue types (Fig. 2*A*). With the emergence of new methods, it is worthwhile to re-examine previous sncRNA profiles that were generated using traditional small RNA sequencing. For instance, PANDORA-seq has enabled novel discoveries about the sncRNA landscape in mature sperm (12, 107), during the reprogramming of somatic cells to induced pluripotent stem cells (12) and in disease conditions such as atherosclerosis (108).

## Not junk

The abundant detection of tsRNAs, rsRNAs, and other sncRNAs derived from longer parental RNAs suggests that these sncRNAs may not be simply random degradation products but are likely to bear functions. Indeed, a closer look into the sequences of these sncRNAs reveals that the detected sequences are much fewer than the theoretical number of all possible fragments from the paternal RNAs (Fig. 2*B*). Additionally, variations in tRNA gene expression at the isodecoder level (tRNAs having the same anticodon sequence) lead to changes in tsRNA, but not in mature tRNA levels (109). This finding suggests that these differences in tRNA gene expression could be unrelated to translation efficiency and instead may play a role in controlled tsRNA biogenesis (109). Moreover, the regions from which the sncRNAs are derived from are not random. For example, some tsRNAs are primarily derived from 5′-halves of tRNA, while others mainly come from 3′-halves (or quarters). Similarly, rsRNAs and other sncRNAs are selectively derived from specific regions of parental rRNAs, showing tissue/cell-type specificity (12, 13) (Fig. 1). These observations collectively underscore the regulatory nature of RNA fragmentation and selective retention.

**Figure 3:**
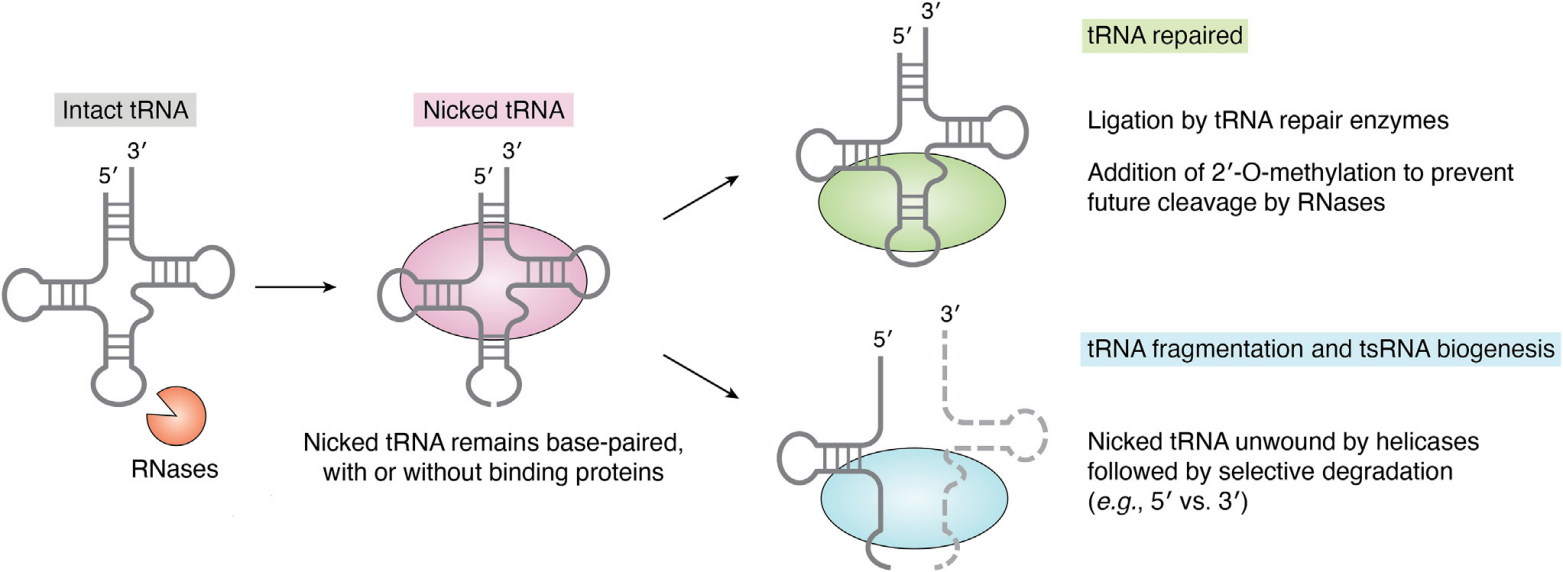
**Enzymatic regulation of tRNA:tsRNA dynamics.** Schematics of tRNA cleavage by RNases, followed by either tRNA repair or selective degradation of tRNA to generate specific tsRNAs. RNase, ribonuclease; tsRNA, tRNA-derived small RNA.

Indeed, recent evidence has demonstrated a stepwise regulation of tsRNA biogenesis. First, tRNAs are nicked by RNases, but the two halves remain base paired and do not separate. This intermediate state may be either maintained by the intrinsic tRNA structure and base-pairing or stabilized by additional binding proteins (110, 111) (Fig. 3). The nicked tRNA can be either repaired with a set of enzymes (110, 111, 112) or further unwound by RNA helicases, leading to the selective degradation of one tRNA half and retention of the other half (113) (Fig. 3). This process of selective cleavage and retention may also be applicable to other types of sncRNAs, governed by the enzymes present in each system, and represent the earliest form of sncRNA biogenesis (1, 5).

Notably, fragmented or nicked tRNAs can be reunited with a set of enzymes in simpler unicellular systems like prokaryotes and yeast (111, 112, 114, 115). This phenomenon may also have connections with the occurrence of split tRNAs observed in some archaeal species (116). In these species, a standard continuous tRNA sequence is divided into two (117) or three (118) separate pieces encoded at different genomic loci, and the fragments of the split tRNAs are individually transcribed and later ligated to form a functional tRNA. Such assembly of small RNA fragments into longer sequences through ligation (by RNA/protein enzymes) could be highly regulated; and may represent an ancient mechanism to build larger RNAs in a primordial world. It remains to be seen whether tsRNA can be reunited into tRNA in mammalian systems under physiological or pathological conditions, a direction that merits future research.

In addition to the highly regulated process of biogenesis, the emerging sncRNAs have shown a range of diverse functions as evidenced from accumulating reports reviewed elsewhere regarding tsRNAs (5, 6, 14, 119, 120, 121, 122, 123), rsRNAs (12, 15), snoRNA-derived small RNAs (28), as well as emerging examples for Y RNA–derived small RNAs (20), vault RNA–derived small RNAs (24, 25, 95), and more. Some of them function through AGO-binding in an RNAi-like fashion and can involve competition between different sncRNAs, such as the case of abnormally increased vault RNA–derived small RNAs (23) or tsRNAs (124) that interfere with the normal siRNA pathways by competitively binding with AGO. In many other cases, sncRNAs can function independently of AGO and sometimes do not even rely on their sequence complementarity to DNA/RNA but through binding with a range of different proteins and/or the formation of ribonucleoproteins (RNPs) (5, 6).

While the reports of RNAi-independent functions for tsRNAs and other sncRNAs continue to grow, these cases have not been generalized to other contexts but are instead often considered exceptions. However, through these cases we perceive new general principles that have the potential to inspire a broader range of possibilities in sncRNA research. We discuss below these principles, which are based on the fundamental properties of sncRNA structure and their aptamer-like interactions with various biological molecules.

## Beyond RNAi

Currently, sncRNAs are mostly known for their RNAi-based functionality, which relies on base pairing with their complementary sequences and recruiting binding proteins (*e.g.*, AGO/PIWI) to act on the target RNA/DNA. However, their other properties such as the potential to fold into various structures and the ability to bind with a wider range of proteins may represent a more fundamental characteristic that is essential for RNAi-independent regulatory functions in tissues and cells.

**Figure 4:**
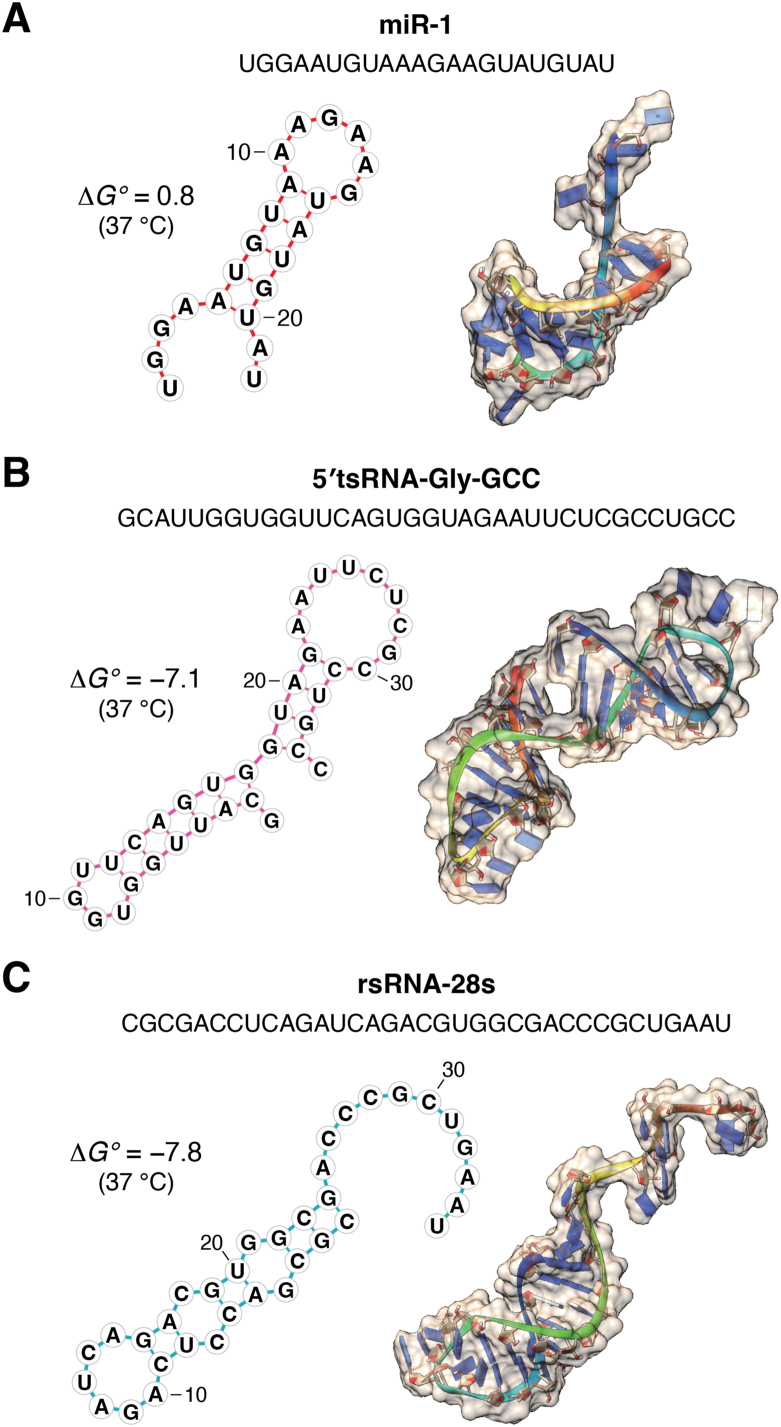
**Predicted secondary and tertiary structures for individual sncRNA sequences.** (*A*) miR-1, (*B*) tsRNA-Gly-GCC, and (*C*) rsRNA-28s. RNAstructure (177) and RNAComposer (178) were used to predict the secondary and tertiary structure of the sncRNAs, respectively. The forna tool (part of ViennaRNA) (179) and UCSF Chimera (180) were used to visualize the secondary and tertiary structure, respectively. The standard free energy difference (*ΔG°* value in kcal/mol) between the folded and unfolded forms of the RNA, predicted at 37 °C, is also presented. Notably, the positive *ΔG°* for miR-1 suggests a preference for unfolded state. Nevertheless, miR-1 has been reported to act as an aptamer by binding with the potassium channel Kir2.1 to change channel activity (135). This suggests that small RNAs can function as aptamers even through their linear form, further underscoring the versatility of aptamer-like functions for small RNAs. rsRNA, rRNA-derived small RNA; sncRNA, small noncoding RNA; tsRNA, tRNA-derived small RNA.

RNA is a highly structured polymer molecule by nature, capable of forming dynamic secondary, tertiary, and higher-order structures. These complex structures can result from self-folding based on minimal free energy (Fig. 4) or interactions between RNA molecules or between RNA and proteins. These structures can interchange and coexist in a dynamic fashion under different environmental conditions (125). The folding and interaction of RNA define its functionality, which ranges from ribozymes and riboswitches to protein binding, as well as the widely studied base pairing with target RNA/DNA sequences (125, 126). When a longer structured RNA (*e.g.*, tRNAs, rRNAs, and snoRNAs) is fragmented into smaller pieces, both the parent and daughter RNAs may be subject to functional selection during evolution. Through this process, numerous possibilities have been tested, with the extant sncRNAs representing the successful outcomes of evolutionary trial and error.

In fact, we argue that the emergence of RNAi only represents a later invention in evolution, which ensures the usage of sncRNAs’ linear sequence for base pairing. In this context, RNAi could be considered a specialized form of sncRNA function, and it may have an origin that predates the need for antiviral activity. We discuss these points below.

## Function as aptamer

The term RNA “aptamer” was first coined in 1990 by Szostak and co-workers (127), which is derived from the Latin *aptus*, meaning fit, and the Greek *meros*, meaning part or region (127, 128). Aptamers are short, single-stranded DNAs or RNAs (typically ranging from 20 to 60 nt) that fold into 3D structures, enabling specific binding to molecular targets of diverse nature, including proteins, metabolites, organic compounds, and even viruses and bacteria (128). In contrast to the RNAi mode of action which primarily depends on linear sequences for base-pair targeting, the function of an RNA aptamer relies on RNA’s secondary and tertiary structures for specific target binding, similar to antibodies.

Notably, while miRNA studies have largely focused on linear sequence-based pairing in an RNAi-like mode, alternative modes of action have occasionally been reported, and can be categorized as exhibiting aptamer-like functions. For instance, let-7, an abundant and evolutionarily conserved miRNA, has been found to function by interacting with toll-like receptor 7 (TLR7) (129), a member of the TLR family that stimulates innate immune responses by recognizing microbial-derived molecules such as lipopolysaccharides, RNA, and DNA (130).

The interaction of let-7 with TLR7 in microglia and macrophages depends on its GU-rich sequence, rather than the 5′ let-7 seed sequence required for posttranscriptional silencing. The binding of let-7 to TLR7 is akin to the GU-rich sequence found in the 20-nt ssRNA40 derived from HIV, a well-known natural RNA activator for TLR7/8 (131). This evidence suggests that let-7 may have an alternative functional mode beyond the classical RNAi-like mechanism.

Interestingly, a more recent study in human monocyte-derived macrophages (HMDMs) discovered that 5′tsRNA-HisGUG can be significantly elevated in HMDMs and their secreted extracellular vesicles upon lipopolysaccharides stimulation, through ANG cleavage (57). The abundance of 5′tsRNA-HisGUG is more than 200 times that of the most abundant miRNAs (*e.g.*, miR-150) in HMDMs, and it can bind to and strongly activate endosomal TLR7 (but not TLR8), thus acting as an immune activator. 5′tsRNA-HisGUG also contains a GU-rich sequence like ssRNA40, suggesting that the GU-rich sequence is a key feature for the binding. However, full-length tRNA-HisGUG cannot stimulate endosomal TLR7 (57), indicating that the binding to TLR7 requires a specific 3D structure of GU-rich sncRNA (*i.e.*, let-7, 5′tsRNA-HisGUG), which is not present when being folded into a tRNA structure. Collectively, although we currently lack specific structural evidence, these examples suggest that let-7 and 5′tsRNA-HisGUG may function in an aptamer-like fashion. Indeed, activation of TLR7/8 by other miRNAs and sncRNAs have also been reported (132, 133), suggesting that it may represent a generalized mechanism for both intracellular and intercellular regulation.

In addition to binding to TLR7, let-7 has also been found to interact with the TRPA1 ion channel to activate nociceptor neurons and elicit pain (134), suggesting that miRNAs can bind to a wider range of functional proteins. In another case, it was discovered that miR-1, a highly conserved miRNA that is abundantly expressed in cardiac and skeletal muscle, can physically bind to Kir2.1, a cardiac membrane potassium channel, and directly regulate channel activity (135). The binding depends on the core sequence of 10A-15G (AAGAAG) in miR-1, which is outside the miRNA seed region. Significantly, a human SNP in miR-1, featuring a A14G mutation, specifically disrupts the miR-1:Kir2.1 interaction while retaining the RNAi function, indicating a structure-based aptamer function of miR-1 independent of RNAi (135).

**Figure 5:**
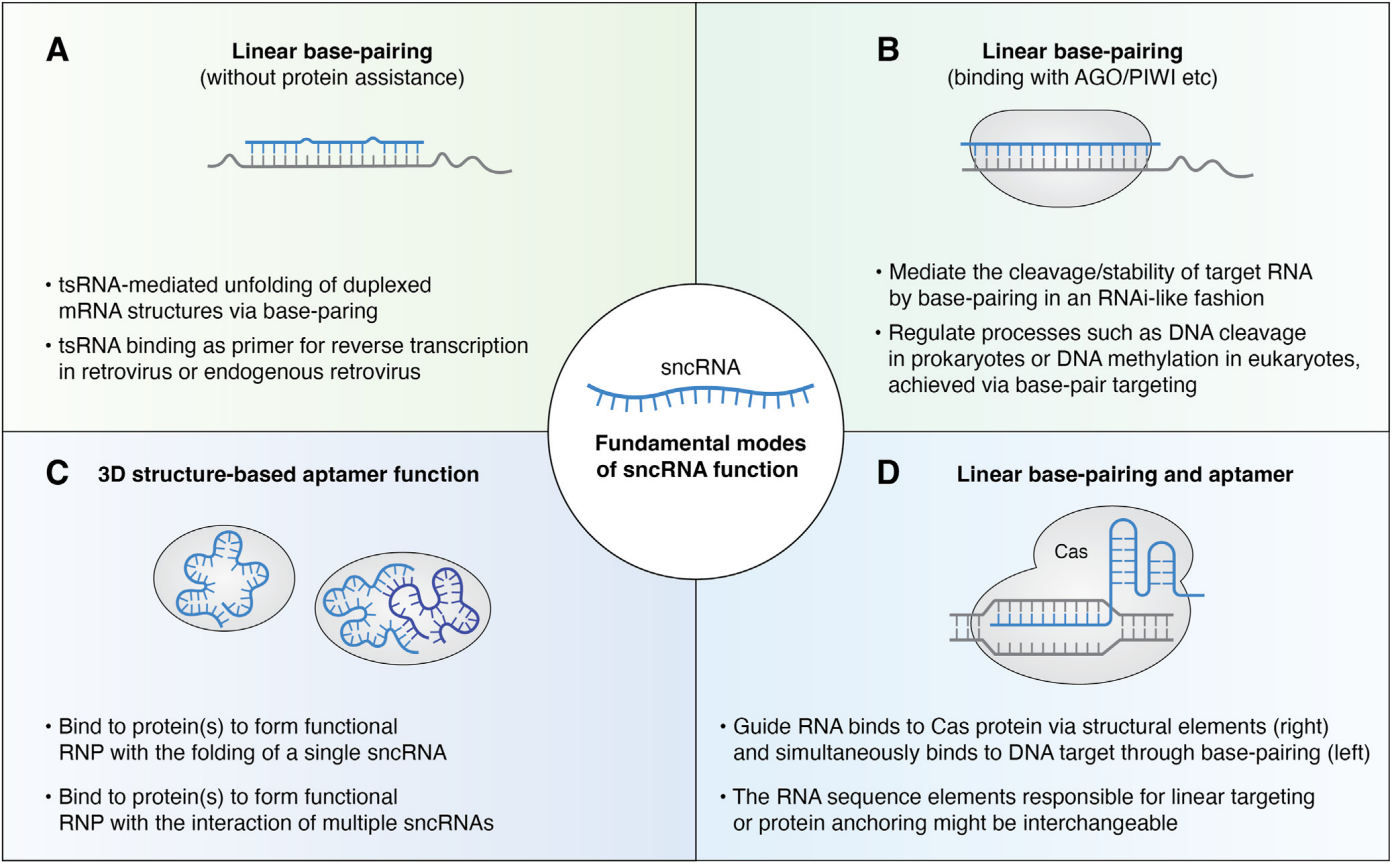
**Different modes of sncRNA function, using linear base pairing, 3D structure-based aptamer function, and a combination of both.***A*, complementarity to target sequence without protein binding. *B*, protein-assisted RNAi-based linear base pairing. *C*, binding with protein using a 3D structure as an aptamer. Note that the sncRNA can fold either on its own or multimerize with other RNAs to form higher-order structures for binding activity. *D*, combination of linear base pairing and aptamer-like function with different domains. For a single sncRNA, the listed functional modes might be interchangeable under different subcellular environments (*e.g.*, cytoplasmic, nuclear, and mitochondrial) and induced by specific local factors. RNP, ribonucleoprotein; sncRNA, small noncoding RNA.

A notable feature in these cases is that the miRNAs and tsRNAs being studied are all abundant in the cell, potentially exceeding the loading capacity of AGO. In fact, it has been reported that the number of miRNAs in the cell exceeds the amount of AGO proteins by more than 13-fold (136), raising an important question regarding the functionality of those miRNAs not bound by AGO. Furthermore, when considering the much greater abundance of sncRNAs (*e.g.*, tsRNAs and rsRNAs) in the cell, they may easily surpass the AGO loading capacity and possibly even the mRNA pool for direct binding. The logical conclusion is that these sncRNAs may either exert base pairing with their target mRNA (and other RNAs) using linear sequence or fold into aptamer structures that exert complementarity-independent functions (Fig. 5, *A* and *C*). Both of these possibilities have been demonstrated. Functions based on AGO-independent base pairing include: (I) tsRNA-mediated unfolding of duplexed mRNA structures, thus regulating translation rates (137, 138) and (II) tsRNA-regulated the RT of retrovirus (or ERV) by either competing or mimicking the effect of natural tRNA as a primer (88, 139, 140). In more cases, the function of sncRNAs could be categorized as aptamer-like, including: (I) regulation of global protein translation (either inhibit or enhance) in unicellular species and prokaryotes by direct tsRNA–ribosome interaction (10, 38, 39, 141); (II) tsRNA-mediated cytoplasmic stress granule formation in regulating mRNA translation and/or the displacement of translational initiation factors (92, 142, 143); (III) tsRNA binding to form nuclear RNPs, which in turn, regulate RNA processing inside the nuclei (144, 145, 146); (IV) tsRNA binding with key RBPs to influence their function in regulating metastatic gene expression in cancer progression (71, 72); (V) tsRNA and other sncRNAs binding with high-density lipoprotein or low-density lipoprotein in the serum for long-range transportation and activation of TLR (132, 147); and (VI) tsRNAs inhibit the association between TSR1 (a ribosome maturation factor) and pre-40S ribosome, leading to a reduction in global protein synthesis (148). In these cases, the sncRNA functions are exerted independent of RNAi to modulate cellular activity, which might be generalized in more situations, especially for other highly expressed sncRNAs.

Importantly, the aptamer-like function of sncRNAs could extend beyond the folded structure of a single sncRNA and may also involve intermolecular structures formed through the interactions between different sncRNAs. For example, in the case of 5′tsRNA-Ala–mediated translational inhibition (92), it has been shown that the displacement of eukaryotic initiation factor 4 gamma to inhibit translational initiation requires the formation of intermolecular RNA-G-quadruplex (RNA-G4) of 5′tsRNA-Ala, a tetramer structure dependent on 5′ terminal oligo-G sequence of 5′tsRNA-Ala (143). Disruption of the RNA-G4 by mutating the continuous 5′ terminal oligo-Gs abolishes the ability of 5′tsRNA-Ala to displace eIF4G (143).

Similarly, stress granule formation induced by 5′tsRNA-Ala also seems to require the RNA-G4 structure (149), consistent with the notion that RNA multimerization driven by both base pairing and tertiary interactions contribute to organization of biomolecular condensates, such as liquid–liquid phase separation (150) (Fig. 5*C*). Finally, just like the function of an RNA aptamer can be improved or modified by incorporating modified nucleotides during their synthesis (151), the naturally occurring RNA modifications on sncRNAs generate a huge reservoir of functionally diverse sncRNAs due to site-specific locations and combinations.

## Rethinking the origin of RNAi

Given that the aptamer-like function of sncRNAs are observed across all domains of life, it is reasonable to argue that it represents a more ancient mode of sncRNA function that predates RNAi. On the other hand, although the aptamer-like function of sncRNAs offers advantages in terms of versatility in target binding, the pool of sequence choices is inherently limited by the size of the genome, as well as the actual transcripts and fragmentation patterns that can be generated in a single cell.

In contrast to the aptamer function, which represents a “default” feature of RNA folding, the emergence of RNAi marks a later evolutionary milestone, which ensures the effective use of sncRNAs’ linear sequences for base pairing–mediated targeting by binding with AGO. This development resulted in a more programmable and simple regulatory system that fully exploits the linear sequences of sncRNAs to bind a diverse range of RNA/DNA targets. In this context, the emergence of RNAi could be considered as a specialized form of an aptamer-like function, wherein AGO binding ensures that sncRNAs do not fold into 3D structures, but maintain a "straightened up" configuration optimal for linear sequence binding (Fig. 5*B*).

The evolutionary origin of RNAi was widely considered to be driven by the defense against transposable elements and RNA viruses (2), thus a case of adaptive evolution. Alternatively, recent considerations argue that RNAi could also have been derived from neutral evolution, emerging as a means of gene regulation before serving as a defense system (152). Our perspective that sncRNA-AGO binding is a specialized form of aptamer function supports the hypothesis of RNAi’s neutral evolutionary origin. It suggests a crucial evolutionary step that ensures sncRNAs’ linear binding with RNA/DNA targets. Notably, the length of this linear-binding region plays a vital role in determining targeting specificity across the genome/transcriptome.

The ancient AGO-like protein may have acquired a catalytic (*e.g.*, RNase H-like) domain capable of DNA/RNA cleavage, forming a prototype of RNAi that may have emerged without the pressure of invading sequences, which initially function in intracellular gene regulation at either the DNA or RNA level. When confronted with invading sequences (DNAs or RNAs), this prototype loaded with linear DNA/RNA may have afforded an advantage for recognition of the invader with sequence complementarily. Further incorporation of specialized components, such as Dicer (only found in eukaryotes (153)), to cleave the invading RNA and load it into AGO-like proteins for effective cleavage of the invader may have led to an efficient defense mechanism. Indeed, many prokaryotes contain pAGO that can load either DNA or RNA to perform target searching, with both catalytically active (154, 155) and catalytically inactive (156) pAGO present.

This proposed origin of RNAi aligns with the dual role of RNAi in both antiviral defense and endogenous gene regulation. We posit that RNAi may have emerged among one of the numerous sncRNA–protein interactions through evolution, parallel to other functional RNPs based on sncRNA–protein interaction. The RNAi modes of action may have been under selection and enhanced in species living in environments with high viral loads, such as soil-dwelling organisms including worms and plants, in which robust and diversified antiviral defense capabilities were demonstrated (2).

Importantly, binding to AGO/PIWI does not necessarily result in RNAi-like functions; AGO/PIWI can also function as delivery proteins for sncRNA to new locations, such as nuclei, where the sncRNAs can function in an aptamer-like fashion. For example, miR-126-5p, an abundant miRNA in endothelial cells, can directly bind and inhibit proapoptotic caspase-3 in the nucleus in an aptamer-like fashion, with nuclear transport of miR-126-5p mediated by a Mex3a/AGO-guided complex (157). This miR-126-5p–mediated inhibition of caspase-3 activity protects endothelial cells and prevention of atherosclerosis (157). In another example in *Tetrahymena*, it has been shown that tsRNA can bind to an AGO/PIWI family protein to form nuclear RNPs, which facilitates nuclear rRNA processing and cell growth (145, 146).

The consideration of sncRNA functionality from an aptamer-centric perspective complements and enhances our understanding of their RNAi functionality. In fact, linear base pairing–mediated DNA/RNA targeting and 3D structure-based binding may coexist or be interchangeable within the same RNA molecules (Fig. 5*D*). For instance, in the CRISPR/Cas system, Cas protein binding with guide RNAs ensures the use of spacer sequences in linear form for base-pair targeting, while the flanking sequences fold in an aptamer-like manner to anchor with the Cas protein (158). Additionally, whether a sncRNA functions by folding on its own or by binding to another RNA sequence with linear complementarity depends on the folding or binding energy it can achieve under each condition. This equilibrium may be further influenced by RNA modifications and local factors such as proteins and metabolites, which can push the equilibrium in either direction. Hence, an expanded view of sncRNA functionality (Fig. 5) that integrates their potential roles as both linear sequence–dependent regulatory molecules and structure-dependent aptamers opens new avenues for investigating their potential roles in various biological processes and diseases.

## Future perspectives

### Challenges and opportunities

The exploration of aptamer-like functions of sncRNAs is still at its infant stage, with great barriers and opportunities lying ahead. One obstacle involves the need for a deeper understanding of sncRNA folding principles and structures, which are inherently more dynamic compared to protein folding (125). For example, one RNA sequence can adopt multiple secondary/tertiary structures with different functions (159, 160, 161, 162, 163). This variability poses potential problems for the commonly used computational prediction of RNA structure based on minimum free energy (Fig. 4), as it may not account for all structural variants and needs experimental validation. Moreover, the situation is further complicated by various RNA modifications. These modifications, known to alter base-pairing rules and influence sncRNA functionality (46, 99), most likely function through the modulation of their structure and binding potential. Given the highly modified nature of tRNA and other structured precursor RNAs (*e.g.*, rRNAs, snRNAs, and snoRNAs) and the dynamic sncRNAs derived from their site-specific cleavage, advanced tools to simultaneously decode sncRNA sequences and site-specific RNA modifications are in great need (1, 164); this challenge is being addressed through the development of mass spectrometry–based approaches (165) as well as nanopore sequencing technology (166). The sncRNA modification information offers both opportunities and also challenge for precise computational and high-throughput AI-based RNA structure prediction, especially as RNA modifications and structures could be dynamically regulated by environment and in subpopulations of RNAs (163, 167).

Nonetheless, the dynamic nature of sncRNA folding and structure does not mean that they are inaccessible. There are molecular interactions and principles that stabilize RNA tertiary structure and thus their functions (126, 168). The functionality of sncRNAs is strongly influenced by interacting molecules, which includes RBPs. Equally important is the subcellular localization and compartmentalization of the sncRNAs in forming biomolecular condensates (150, 169). Understanding these principles using advanced methods in probing RNA structures (170) under different biological contexts will promote the computational prediction of RNA–protein interaction and eventually lead to the deciphering of their functionality.

### Explore with caution

While the RNAi system offers elegant simplicity for sncRNA-based gene regulation, understanding other functional principles of sncRNAs, such as those in an aptamer-centric view, provides a more holistic perspective on the complexity of sncRNA functionality. At times, the inherent complexity of these systems becomes the very source of their order, giving rise to the cells and organisms which we observe today.

The modern pharmaceutical industry largely relies on a reductionist approach, simplifying complex systems to harness key principles for treating diseases. This approach is evident in the successful application of the well-defined RNAi mechanism in creating new therapeutics based on targeted gene silencing through sequence-complementarity, as pioneered by Alnylam Therapeutics (171). However, the development of therapeutics that targets miRNAs–such as antagomirs or anti-miRs (chemically engineered antisense oligonucleotides designed to target and silence specific endogenous miRNAs based on sequence complementarity)–has not been as fruitful despite substantial investment (172, 173). This failure may reflect the multifaceted function of highly expressed miRNAs: they can both exert RNAi through linear miRNA–mRNA base pairing and aptamer function through miRNA–protein interactions. An antagomir might successfully block the RNAi portion of miRNA activity, but not the portion with folded structures that remain bound to proteins. This limitation could potentially be overcome by using small molecules that target specific RNA structures (174), a strategy that would be informed by future investigations of RNA structural information. Moreover, when the delivered antagomirs saturate their linear sequence complementarity-based binding targets, they may fold into unexpected structures and exert aptamer-like functions, causing unexplored “off-target” side effects. In fact, the folding features of other sncRNA mimics (*e.g.*, agomir) have similar concerns, potentially generating nonspecific effects (175) through unexpected RNA–RNA interactions and aptamer effects.

Now, as we delve deeper into the vast sncRNA universe beyond miRNAs (Figs. 1 and 2), it becomes imperative to embrace a broader spectrum of their functional principles. This exploration requires a step beyond the “information cocoon” that has previously shaped our understanding of sncRNA functionality, a perspective that has been predominantly centered around simple complementarity–based mechanisms. Otherwise, the pursuit of simplicity risks overlooking the profound complexity intrinsic to the system, a complexity we refer to as the “RNA code” (1, 176). After all, as stated by the great philosopher Alfred North Whitehead, *“...the only simplicity to be trusted is the simplicity to be found on the far side of complexity.*”

## Conflict of interest

The authors declare that they have no conflicts of interest with the contents of this article.

## References

[1] Shi J., Zhou T., Chen Q. (2022). Exploring the expanding universe of small RNAs. Nat. Cell Biol..

[2] Carthew R.W., Sontheimer E.J. (2009). Origins and mechanisms of miRNAs and siRNAs. Cell.

[3] Bartel D.P. (2018). Metazoan MicroRNAs. Cell.

[4] Ozata D.M., Gainetdinov I., Zoch A., O’Carroll D., Zamore P.D. (2019). PIWI-interacting RNAs: small RNAs with big functions. Nat. Rev. Genet..

[5] Chen Q., Zhang X., Shi J., Yan M., Zhou T. (2021). Origins and evolving functionalities of tRNA-derived small RNAs. Trends Biochem. Sci..

[6] Su Z., Wilson B., Kumar P., Dutta A. (2020). Noncanonical roles of tRNAs: tRNA fragments and beyond. Annu. Rev. Genet..

[7] Holmes A.D., Chan P.P., Chen Q., Ivanov P., Drouard L., Polacek N. (2023). A standardized ontology for naming tRNA-derived RNAs based on molecular origin. Nat. Methods.

[8] Raad N., Luidalepp H., Fasnacht M., Polacek N. (2021). Transcriptome-wide analysis of stationary phase small ncRNAs in E. coli. Int. J. Mol. Sci..

[9] Thompson D.M., Lu C., Green P.J., Parker R. (2008). tRNA cleavage is a conserved response to oxidative stress in eukaryotes. RNA.

[10] Gebetsberger J., Zywicki M., Kunzi A., Polacek N. (2012). tRNA-derived fragments target the ribosome and function as regulatory non-coding RNA in Haloferax volcanii. Archaea.

[11] Tyczewska A., Grzywacz K. (2022). tRNA-derived fragments as new players in regulatory processes in yeast. Yeast.

[12] Shi J., Zhang Y., Tan D., Zhang X., Yan M., Zhang Y. (2021). PANDORA-seq expands the repertoire of regulatory small RNAs by overcoming RNA modifications. Nat. Cell Biol..

[13] Wang H., Huang R., Li L., Zhu J., Li Z., Peng C. (2021). CPA-seq reveals small ncRNAs with methylated nucleosides and diverse termini. Cell Discov..

[14] Schimmel P. (2018). The emerging complexity of the tRNA world: mammalian tRNAs beyond protein synthesis. Nat. Rev. Mol. Cell Biol..

[15] Lambert M., Benmoussa A., Provost P. (2019). Small non-coding RNAs derived from eukaryotic ribosomal RNA. Noncoding RNA.

[16] Shi J., Ko E.A., Sanders K.M., Chen Q., Zhou T. (2018). SPORTS1.0: a tool for annotating and profiling non-coding RNAs Optimized for rRNA- and tRNA-derived small RNAs. Genomics Proteomics Bioinformatics.

[17] Taft R.J., Glazov E.A., Lassmann T., Hayashizaki Y., Carninci P., Mattick J.S. (2009). Small RNAs derived from snoRNAs. RNA.

[18] Ender C., Krek A., Friedlander M.R., Beitzinger M., Weinmann L., Chen W. (2008). A human snoRNA with microRNA-like functions. Mol. Cell.

[19] Chen C.J., Heard E. (2013). Small RNAs derived from structural non-coding RNAs. Methods.

[20] Cambier L., de Couto G., Ibrahim A., Echavez A.K., Valle J., Liu W. (2017). Y RNA fragment in extracellular vesicles confers cardioprotection via modulation of IL-10 expression and secretion. EMBO Mol. Med..

[21] Gu W., Shi J., Liu H., Zhang X., Zhou J.J., Li M. (2020). Peripheral blood non-canonical small non-coding RNAs as novel biomarkers in lung cancer. Mol. Cancer.

[22] Persson H., Kvist A., Vallon-Christersson J., Medstrand P., Borg A., Rovira C. (2009). The non-coding RNA of the multidrug resistance-linked vault particle encodes multiple regulatory small RNAs. Nat. Cell Biol..

[23] Hussain S., Sajini A.A., Blanco S., Dietmann S., Lombard P., Sugimoto Y. (2013). NSun2-mediated cytosine-5 methylation of vault noncoding RNA determines its processing into regulatory small RNAs. Cell Rep..

[24] Sajini A.A., Choudhury N.R., Wagner R.E., Bornelov S., Selmi T., Spanos C. (2019). Loss of 5-methylcytosine alters the biogenesis of vault-derived small RNAs to coordinate epidermal differentiation. Nat. Commun..

[25] Alagia A., Terenova J., Ketley R.F., Di Fazio A., Chelysheva I., Gullerova M. (2023). Small vault RNA1-2 modulates expression of cell membrane proteins through nascent RNA silencing. Life Sci. Alliance.

[26] Pircher A., Bakowska-Zywicka K., Schneider L., Zywicki M., Polacek N. (2014). An mRNA-derived noncoding RNA targets and regulates the ribosome. Mol. Cell.

[27] Reuther J., Schneider L., Iacovache I., Pircher A., Gharib W.H., Zuber B. (2021). A small ribosome-associated ncRNA globally inhibits translation by restricting ribosome dynamics. RNA Biol..

[28] Wajahat M., Bracken C.P., Orang A. (2021). Emerging functions for snoRNAs and snoRNA-derived fragments. Int. J. Mol. Sci..

[29] Mattick J., Amaral P. (2023).

[30] Marx V. (2022). How noncoding RNAs began to leave the junkyard. Nat. Methods.

[31] Lee S.R., Collins K. (2005). Starvation-induced cleavage of the tRNA anticodon loop in Tetrahymena thermophila. J. Biol. Chem..

[32] Li Y., Luo J., Zhou H., Liao J.Y., Ma L.M., Chen Y.Q. (2008). Stress-induced tRNA-derived RNAs: a novel class of small RNAs in the primitive eukaryote Giardia lamblia. Nucleic Acids Res..

[33] Haussecker D., Huang Y., Lau A., Parameswaran P., Fire A.Z., Kay M.A. (2010). Human tRNA-derived small RNAs in the global regulation of RNA silencing. RNA.

[34] Yamasaki S., Ivanov P., Hu G.F., Anderson P. (2009). Angiogenin cleaves tRNA and promotes stress-induced translational repression. J. Cell Biol..

[35] Lee Y.S., Shibata Y., Malhotra A., Dutta A. (2009). A novel class of small RNAs: tRNA-derived RNA fragments (tRFs). Genes Dev..

[36] Fu H., Feng J., Liu Q., Sun F., Tie Y., Zhu J. (2009). Stress induces tRNA cleavage by angiogenin in mammalian cells. FEBS Lett..

[37] Garcia-Silva M.R., das Neves R.F., Cabrera-Cabrera F., Sanguinetti J., Medeiros L.C., Robello C. (2014). Extracellular vesicles shed by Trypanosoma cruzi are linked to small RNA pathways, life cycle regulation, and susceptibility to infection of mammalian cells. Parasitol. Res..

[38] Gebetsberger J., Wyss L., Mleczko A.M., Reuther J., Polacek N. (2017). A tRNA-derived fragment competes with mRNA for ribosome binding and regulates translation during stress. RNA Biol..

[39] Fricker R., Brogli R., Luidalepp H., Wyss L., Fasnacht M., Joss O. (2019). A tRNA half modulates translation as stress response in Trypanosoma brucei. Nat. Commun..

[40] Sharma M., Zhang H., Ehrenkaufer G., Singh U. (2023). Stress response in Entamoeba histolytica is associated with robust processing of tRNA to tRNA halves. mBio.

[41] Thompson D.M., Parker R. (2009). The RNase Rny1p cleaves tRNAs and promotes cell death during oxidative stress in Saccharomyces cerevisiae. J. Cell Biol..

[42] Saikia M., Krokowski D., Guan B.J., Ivanov P., Parisien M., Hu G.F. (2012). Genome-wide identification and quantitative analysis of cleaved tRNA fragments induced by cellular stress. J. Biol. Chem..

[43] Hasler D., Lehmann G., Murakawa Y., Klironomos F., Jakob L., Grasser F.A. (2016). The Lupus autoantigen La prevents Mis-channeling of tRNA fragments into the human MicroRNA pathway. Mol. Cell.

[44] Tuorto F., Liebers R., Musch T., Schaefer M., Hofmann S., Kellner S. (2012). RNA cytosine methylation by Dnmt2 and NSun2 promotes tRNA stability and protein synthesis. Nat. Struct. Mol. Biol..

[45] Schaefer M., Pollex T., Hanna K., Tuorto F., Meusburger M., Helm M. (2010). RNA methylation by Dnmt2 protects transfer RNAs against stress-induced cleavage. Genes Dev..

[46] Zhang Y., Zhang X., Shi J., Tuorto F., Li X., Liu Y. (2018). Dnmt2 mediates intergenerational transmission of paternally acquired metabolic disorders through sperm small non-coding RNAs. Nat. Cell Biol..

[47] Blanco S., Bandiera R., Popis M., Hussain S., Lombard P., Aleksic J. (2016). Stem cell function and stress response are controlled by protein synthesis. Nature.

[48] Zhang X., Cozen A.E., Liu Y., Chen Q., Lowe T.M. (2016). Small RNA modifications: integral to function and disease. Trends Mol. Med..

[49] Pichot F., Hogg M.C., Marchand V., Bourguignon V., Jirstrom E., Farrell C. (2023). Quantification of substoichiometric modification reveals global tsRNA hypomodification, preferences for angiogenin-mediated tRNA cleavage, and idiosyncratic epitranscriptomes of human neuronal cell-lines. Comput. Struct. Biotechnol. J..

[50] Watkins C.P., Zhang W., Wylder A.C., Katanski C.D., Pan T. (2022). A multiplex platform for small RNA sequencing elucidates multifaceted tRNA stress response and translational regulation. Nat. Commun..

[51] Peng H., Shi J., Zhang Y., Zhang H., Liao S., Li W. (2012). A novel class of tRNA-derived small RNAs extremely enriched in mature mouse sperm. Cell Res..

[52] Chen Q., Yan M., Cao Z., Li X., Zhang Y., Shi J. (2016). Sperm tsRNAs contribute to intergenerational inheritance of an acquired metabolic disorder. Science.

[53] Sharma U., Conine C.C., Shea J.M., Boskovic A., Derr A.G., Bing X.Y. (2016). Biogenesis and function of tRNA fragments during sperm maturation and fertilization in mammals. Science.

[54] Zhang Y., Zhang Y., Shi J., Zhang H., Cao Z., Gao X. (2014). Identification and characterization of an ancient class of small RNAs enriched in serum associating with active infection. J. Mol. Cell Biol..

[55] Dhahbi J.M., Spindler S.R., Atamna H., Yamakawa A., Boffelli D., Mote P. (2013). 5' tRNA halves are present as abundant complexes in serum, concentrated in blood cells, and modulated by aging and calorie restriction. BMC Genomics.

[56] Godoy P.M., Bhakta N.R., Barczak A.J., Cakmak H., Fisher S., MacKenzie T.C. (2018). Large differences in small RNA composition between human biofluids. Cell Rep..

[57] Pawar K., Shigematsu M., Sharbati S., Kirino Y. (2020). Infection-induced 5'-half molecules of tRNAHisGUG activate Toll-like receptor 7. PLoS Biol..

[58] Tosar J.P., Segovia M., Castellano M., Gambaro F., Akiyama Y., Fagundez P. (2020). Fragmentation of extracellular ribosomes and tRNAs shapes the extracellular RNAome. Nucleic Acids Res..

[59] Shurtleff M.J., Yao J., Qin Y., Nottingham R.M., Temoche-Diaz M.M., Schekman R. (2017). Broad role for YBX1 in defining the small noncoding RNA composition of exosomes. Proc. Natl. Acad. Sci. U. S. A..

[60] Nechooshtan G., Yunusov D., Chang K., Gingeras T.R. (2020). Processing by RNase 1 forms tRNA halves and distinct Y RNA fragments in the extracellular environment. Nucleic Acids Res..

[61] Kfoury Y.S., Ji F., Mazzola M., Sykes D.B., Scherer A.K., Anselmo A. (2021). tiRNA signaling via stress-regulated vesicle transfer in the hematopoietic niche. Cell Stem Cell.

[62] Hogg M.C., Raoof R., El Naggar H., Monsefi N., Delanty N., O'Brien D.F. (2019). Elevation in plasma tRNA fragments precede seizures in human epilepsy. J. Clin. Invest..

[63] Paris Z., Alfonzo J.D. (2018). How the intracellular partitioning of tRNA and tRNA modification enzymes affects mitochondrial function. IUBMB Life.

[64] Balatti V., Nigita G., Veneziano D., Drusco A., Stein G.S., Messier T.L. (2017). tsRNA signatures in cancer. Proc. Natl. Acad. Sci. U. S. A..

[65] Zhan S., Yang P., Zhou S., Xu Y., Xu R., Liang G. (2022). Serum mitochondrial tsRNA serves as a novel biomarker for hepatocarcinoma diagnosis. Front. Med..

[66] Zhang X., Trebak F., Souza L.A.C., Shi J., Zhou T., Kehoe P.G. (2020). Small RNA modifications in Alzheimer's disease. Neurobiol. Dis..

[67] Zhu L., Li J., Gong Y., Wu Q., Tan S., Sun D. (2019). Exosomal tRNA-derived small RNA as a promising biomarker for cancer diagnosis. Mol. Cancer.

[68] Chen X., Sun Q., Zheng Y., Liu Z., Meng X., Zeng W. (2021). Human sperm tsRNA as potential biomarker and therapy target for male fertility. Reproduction.

[69] Nunes A., Ribeiro D.R., Marques M., Santos M.A.S., Ribeiro D., Soares A.R. (2020). Emerging roles of tRNAs in RNA virus infections. Trends Biochem. Sci..

[70] Wang Q., Lee I., Ren J., Ajay S.S., Lee Y.S., Bao X. (2013). Identification and functional characterization of tRNA-derived RNA fragments (tRFs) in respiratory syncytial virus infection. Mol. Ther..

[71] Goodarzi H., Liu X., Nguyen H.C., Zhang S., Fish L., Tavazoie S.F. (2015). Endogenous tRNA-derived fragments suppress breast cancer progression via YBX1 displacement. Cell.

[72] Liu X., Mei W., Padmanaban V., Alwaseem H., Molina H., Passarelli M.C. (2022). A pro-metastatic tRNA fragment drives Nucleolin oligomerization and stabilization of its bound metabolic mRNAs. Mol. Cell.

[73] Honda S., Loher P., Shigematsu M., Palazzo J.P., Suzuki R., Imoto I. (2015). Sex hormone-dependent tRNA halves enhance cell proliferation in breast and prostate cancers. Proc. Natl. Acad. Sci. U. S. A..

[74] Krishna S., Yim D.G., Lakshmanan V., Tirumalai V., Koh J.L., Park J.E. (2019). Dynamic expression of tRNA-derived small RNAs define cellular states. EMBO Rep..

[75] Guzzi N., Ciesla M., Ngoc P.C.T., Lang S., Arora S., Dimitriou M. (2018). Pseudouridylation of tRNA-derived fragments Steers translational Control in stem cells. Cell.

[76] Sarker G., Sun W., Rosenkranz D., Pelczar P., Opitz L., Efthymiou V. (2019). Maternal overnutrition programs hedonic and metabolic phenotypes across generations through sperm tsRNAs. Proc. Natl. Acad. Sci. U. S. A..

[77] Zhang Y., Ren L., Sun X., Zhang Z., Liu J., Xin Y. (2021). Angiogenin mediates paternal inflammation-induced metabolic disorders in offspring through sperm tsRNAs. Nat. Commun..

[78] Alata Jimenez N., Castellano M., Santillan E.M., Boulias K., Boan A., Arias Padilla L.F. (2023). Paternal methotrexate exposure affects sperm small RNA content and causes craniofacial defects in the offspring. Nat. Commun..

[79] Chen Q., Yan W., Duan E. (2016). Epigenetic inheritance of acquired traits through sperm RNAs and sperm RNA modifications. Nat. Rev. Genet..

[80] Diallo I., Ho J., Lambert M., Benmoussa A., Husseini Z., Lalaouna D. (2022). A tRNA-derived fragment present in E. coli OMVs regulates host cell gene expression and proliferation. PLoS Pathog..

[81] Ren B., Wang X., Duan J., Ma J. (2019). Rhizobial tRNA-derived small RNAs are signal molecules regulating plant nodulation. Science.

[82] Kuscu C., Kumar P., Kiran M., Su Z., Malik A., Dutta A. (2018). tRNA fragments (tRFs) guide Ago to regulate gene expression post-transcriptionally in a Dicer-independent manner. RNA.

[83] Maute R.L., Schneider C., Sumazin P., Holmes A., Califano A., Basso K. (2013). tRNA-derived microRNA modulates proliferation and the DNA damage response and is down-regulated in B cell lymphoma. Proc. Natl. Acad. Sci. U. S. A..

[84] Luo S., He F., Luo J., Dou S., Wang Y., Guo A. (2018). Drosophila tsRNAs preferentially suppress general translation machinery via antisense pairing and participate in cellular starvation response. Nucleic Acids Res..

[85] Sobala A., Hutvagner G. (2013). Small RNAs derived from the 5' end of tRNA can inhibit protein translation in human cells. RNA Biol..

[86] Su Z., Kuscu C., Malik A., Shibata E., Dutta A. (2019). Angiogenin generates specific stress-induced tRNA halves and is not involved in tRF-3-mediated gene silencing. J. Biol. Chem..

[87] Cho H., Lee W., Kim G.W., Lee S.H., Moon J.S., Kim M. (2019). Regulation of La/SSB-dependent viral gene expression by pre-tRNA 3' trailer-derived tRNA fragments. Nucleic Acids Res..

[88] Schorn A.J., Gutbrod M.J., LeBlanc C., Martienssen R. (2017). LTR-retrotransposon Control by tRNA-derived small RNAs. Cell.

[89] Sim G., Kehling A.C., Park M.S., Secor J., Divoky C., Zhang H. (2022). Manganese-dependent microRNA trimming by 3'-->5' exonucleases generates 14-nucleotide or shorter tiny RNAs. Proc. Natl. Acad. Sci. U. S. A..

[90] Park M.S., Sim G., Kehling A.C., Nakanishi K. (2020). Human Argonaute2 and Argonaute3 are catalytically activated by different lengths of guide RNA. Proc. Natl. Acad. Sci. U. S. A..

[91] Nakanishi K. (2022). Anatomy of four human Argonaute proteins. Nucleic Acids Res..

[92] Ivanov P., Emara M.M., Villen J., Gygi S.P., Anderson P. (2011). Angiogenin-induced tRNA fragments inhibit translation initiation. Mol. Cell.

[93] Sanadgol N., Konig L., Drino A., Jovic M., Schaefer M.R. (2022). Experimental paradigms revisited: oxidative stress-induced tRNA fragmentation does not correlate with stress granule formation but is associated with delayed cell death. Nucleic Acids Res..

[94] Lee H.C., Chang S.S., Choudhary S., Aalto A.P., Maiti M., Bamford D.H. (2009). qiRNA is a new type of small interfering RNA induced by DNA damage. Nature.

[95] Hahne J.C., Lampis A., Valeri N. (2021). Vault RNAs: hidden gems in RNA and protein regulation. Cell. Mol. Life Sci..

[96] Suzuki T. (2021). The expanding world of tRNA modifications and their disease relevance. Nat. Rev. Mol. Cell Biol..

[97] Boccaletto P., Stefaniak F., Ray A., Cappannini A., Mukherjee S., Purta E. (2022). MODOMICS: a database of RNA modification pathways. 2021 update. Nucleic Acids Res..

[98] Lei H.T., Wang Z.H., Li B., Sun Y., Mei S.Q., Yang J.H. (2023). tModBase: deciphering the landscape of tRNA modifications and their dynamic changes from epitranscriptome data. Nucleic Acids Res..

[99] Su Z., Monshaugen I., Wilson B., Wang F., Klungland A., Ougland R. (2022). TRMT6/61A-dependent base methylation of tRNA-derived fragments regulates gene-silencing activity and the unfolded protein response in bladder cancer. Nat. Commun..

[100] Zheng G., Qin Y., Clark W.C., Dai Q., Yi C., He C. (2015). Efficient and quantitative high-throughput tRNA sequencing. Nat. Methods.

[101] Cozen A.E., Quartley E., Holmes A.D., Hrabeta-Robinson E., Phizicky E.M., Lowe T.M. (2015). ARM-seq: AlkB-facilitated RNA methylation sequencing reveals a complex landscape of modified tRNA fragments. Nat. Methods.

[102] Xu H., Yao J., Wu D.C., Lambowitz A.M. (2019). Improved TGIRT-seq methods for comprehensive transcriptome profiling with decreased adapter dimer formation and bias correction. Sci. Rep..

[103] Upton H.E., Ferguson L., Temoche-Diaz M.M., Liu X.M., Pimentel S.C., Ingolia N.T. (2021). Low-bias ncRNA libraries using ordered two-template relay: serial template jumping by a modified retroelement reverse transcriptase. Proc. Natl. Acad. Sci. U. S. A..

[104] Hu J.F., Yim D., Ma D., Huber S.M., Davis N., Bacusmo J.M. (2021). Quantitative mapping of the cellular small RNA landscape with AQRNA-seq. Nat. Biotechnol..

[105] Scheepbouwer C., Aparicio-Puerta E., Gomez-Martin C., Verschueren H., van Eijndhoven M., Wedekind L.E. (2023). ALL-tRNAseq enables robust tRNA profiling in tissue samples. Genes Dev..

[106] Behrens A., Rodschinka G., Nedialkova D.D. (2021). High-resolution quantitative profiling of tRNA abundance and modification status in eukaryotes by mim-tRNAseq. Mol. Cell.

[107] Chen Q. (2022). Sperm RNA-mediated epigenetic inheritance in mammals: challenges and opportunities. Reprod. Fertil. Dev..

[108] Hernandez R., Shi J., Liu J., Li X., Wu J., Zhao L. (2023). PANDORA-seq unveils the hidden small non-coding RNA landscape in atherosclerosis of LDL receptor-deficient mice. J. Lipid Res..

[109] Torres A.G., Reina O., Stephan-Otto Attolini C., Ribas de Pouplana L. (2019). Differential expression of human tRNA genes drives the abundance of tRNA-derived fragments. Proc. Natl. Acad. Sci. U. S. A..

[110] Costa B., Li Calzi M., Castellano M., Blanco V., Cuevasanta E., Litvan I. (2023). Nicked tRNAs are stable reservoirs of tRNA halves in cells and biofluids. Proc. Natl. Acad. Sci. U. S. A..

[111] Chen X., Wolin S.L. (2023). Transfer RNA halves are found as nicked tRNAs in cells: evidence that nicked tRNAs regulate expression of an RNA repair operon. RNA.

[112] Chan C.M., Zhou C., Huang R.H. (2009). Reconstituting bacterial RNA repair and modification *in vitro*. Science.

[113] Drino A., Konig L., Capitanchik C., Sanadgol N., Janisiw E., Rappol T. (2023). Identification of RNA helicases with unwinding activity on angiogenin-processed tRNAs. Nucleic Acids Res..

[114] Hughes K.J., Chen X., Burroughs A.M., Aravind L., Wolin S.L. (2020). An RNA repair operon regulated by Damaged tRNAs. Cell Rep..

[115] Phizicky E.M., Hopper A.K. (2023). The life and times of a tRNA. RNA.

[116] Chan P.P., Cozen A.E., Lowe T.M. (2011). Discovery of permuted and recently split transfer RNAs in Archaea. Genome Biol..

[117] Randau L., Munch R., Hohn M.J., Jahn D., Soll D. (2005). Nanoarchaeum equitans creates functional tRNAs from separate genes for their 5'- and 3'-halves. Nature.

[118] Fujishima K., Sugahara J., Kikuta K., Hirano R., Sato A., Tomita M. (2009). Tri-split tRNA is a transfer RNA made from 3 transcripts that provides insight into the evolution of fragmented tRNAs in archaea. Proc. Natl. Acad. Sci. U. S. A..

[119] Shi J., Zhang Y., Zhou T., Chen Q. (2019). tsRNAs: the Swiss Army Knife for translational regulation. Trends Biochem. Sci..

[120] Oberbauer V., Schaefer M.R. (2018). tRNA-derived small RNAs: biogenesis, modification, function and potential Impact on human disease development. Genes (Basel).

[121] Anderson P., Ivanov P. (2014). tRNA fragments in human health and disease. FEBS Lett..

[122] Kumar P., Kuscu C., Dutta A. (2016). Biogenesis and function of transfer RNA-Related fragments (tRFs). Trends Biochem. Sci..

[123] Magee R., Rigoutsos I. (2020). On the expanding roles of tRNA fragments in modulating cell behavior. Nucleic Acids Res..

[124] Durdevic Z., Mobin M.B., Hanna K., Lyko F., Schaefer M. (2013). The RNA methyltransferase Dnmt2 is required for efficient Dicer-2-dependent siRNA pathway activity in Drosophila. Cell Rep..

[125] Ganser L.R., Kelly M.L., Herschlag D., Al-Hashimi H.M. (2019). The roles of structural dynamics in the cellular functions of RNAs. Nat. Rev. Mol. Cell Biol..

[126] Assmann S.M., Chou H.L., Bevilacqua P.C. (2023). Rock, scissors, paper: how RNA structure informs function. Plant Cell.

[127] Ellington A.D., Szostak J.W. (1990). *In vitro* selection of RNA molecules that bind specific ligands. Nature.

[128] Zhou J., Rossi J. (2017). Aptamers as targeted therapeutics: current potential and challenges. Nat. Rev. Drug Discov..

[129] Lehmann S.M., Kruger C., Park B., Derkow K., Rosenberger K., Baumgart J. (2012). An unconventional role for miRNA: let-7 activates Toll-like receptor 7 and causes neurodegeneration. Nat. Neurosci..

[130] O'Neill L.A., Golenbock D., Bowie A.G. (2013). The history of Toll-like receptors - redefining innate immunity. Nat. Rev. Immunol..

[131] Heil F., Hemmi H., Hochrein H., Ampenberger F., Kirschning C., Akira S. (2004). Species-specific recognition of single-stranded RNA via toll-like receptor 7 and 8. Science.

[132] Allen R.M., Michell D.L., Cavnar A.B., Zhu W., Makhijani N., Contreras D.M. (2022). LDL delivery of microbial small RNAs drives atherosclerosis through macrophage TLR8. Nat. Cell Biol..

[133] Fabbri M., Paone A., Calore F., Galli R., Gaudio E., Santhanam R. (2012). MicroRNAs bind to Toll-like receptors to induce prometastatic inflammatory response. Proc. Natl. Acad. Sci. U. S. A..

[134] Park C.K., Xu Z.Z., Berta T., Han Q., Chen G., Liu X.J. (2014). Extracellular microRNAs activate nociceptor neurons to elicit pain via TLR7 and TRPA1. Neuron.

[135] Yang D., Wan X., Dennis A.T., Bektik E., Wang Z., Costa M.G.S. (2021). MicroRNA Biophysically modulates cardiac action potential by direct binding to ion channel. Circulation.

[136] Janas M.M., Wang B., Harris A.S., Aguiar M., Shaffer J.M., Subrahmanyam Y.V. (2012). Alternative RISC assembly: binding and repression of microRNA-mRNA duplexes by human Ago proteins. RNA.

[137] Kim H.K., Fuchs G., Wang S., Wei W., Zhang Y., Park H. (2017). A transfer-RNA-derived small RNA regulates ribosome biogenesis. Nature.

[138] Kim H.K., Xu J., Chu K., Park H., Jang H., Li P. (2019). A tRNA-derived small RNA regulates ribosomal protein S28 protein levels after translation initiation in humans and mice. Cell Rep..

[139] Ruggero K., Guffanti A., Corradin A., Sharma V.K., De Bellis G., Corti G. (2014). Small noncoding RNAs in cells transformed by human T-cell leukemia virus type 1: a role for a tRNA fragment as a primer for reverse transcriptase. J. Virol..

[140] Yeung M.L., Bennasser Y., Watashi K., Le S.Y., Houzet L., Jeang K.T. (2009). Pyrosequencing of small non-coding RNAs in HIV-1 infected cells: evidence for the processing of a viral-cellular double-stranded RNA hybrid. Nucleic Acids Res..

[141] Gonskikh Y., Gerstl M., Kos M., Borth N., Schosserer M., Grillari J. (2020). Modulation of mammalian translation by a ribosome-associated tRNA half. RNA Biol..

[142] Emara M.M., Ivanov P., Hickman T., Dawra N., Tisdale S., Kedersha N. (2010). Angiogenin-induced tRNA-derived stress-induced RNAs promote stress-induced stress granule assembly. J. Biol. Chem..

[143] Lyons S.M., Kharel P., Akiyama Y., Ojha S., Dave D., Tsvetkov V. (2020). eIF4G has intrinsic G-quadruplex binding activity that is required for tiRNA function. Nucleic Acids Res..

[144] Boskovic A., Bing X.Y., Kaymak E., Rando O.J. (2020). Control of noncoding RNA production and histone levels by a 5' tRNA fragment. Genes Dev..

[145] Couvillion M.T., Bounova G., Purdom E., Speed T.P., Collins K. (2012). A Tetrahymena Piwi bound to mature tRNA 3' fragments activates the exonuclease Xrn2 for RNA processing in the nucleus. Mol. Cell.

[146] Couvillion M.T., Sachidanandam R., Collins K. (2010). A growth-essential Tetrahymena Piwi protein carries tRNA fragment cargo. Genes Dev..

[147] Michell D.L., Allen R.M., Cavnar A.B., Contreras D.M., Yu M., Semler E.M. (2022). Elucidation of physico-chemical principles of high-density lipoprotein-small RNA binding interactions. J. Biol. Chem..

[148] Ying S., Li P., Wang J., Chen K., Zou Y., Dai M. (2023). tRF-Gln-CTG-026 ameliorates liver injury by alleviating global protein synthesis. Signal Transduct. Target. Ther..

[149] Lyons S.M., Gudanis D., Coyne S.M., Gdaniec Z., Ivanov P. (2017). Identification of functional tetramolecular RNA G-quadruplexes derived from transfer RNAs. Nat. Commun..

[150] Bevilacqua P.C., Williams A.M., Chou H.L., Assmann S.M. (2022). RNA multimerization as an organizing force for liquid-liquid phase separation. RNA.

[151] Ji D., Feng H., Liew S.W., Kwok C.K. (2023). Modified nucleic acid aptamers: development, characterization, and biological applications. Trends Biotechnol..

[152] Torri A., Jaeger J., Pradeu T., Saleh M.C. (2022). The origin of RNA interference: adaptive or neutral evolution?. PLoS Biol..

[153] Shabalina S.A., Koonin E.V. (2008). Origins and evolution of eukaryotic RNA interference. Trends Ecol. Evol..

[154] Swarts D.C., Jore M.M., Westra E.R., Zhu Y., Janssen J.H., Snijders A.P. (2014). DNA-guided DNA interference by a prokaryotic Argonaute. Nature.

[155] Kaya E., Doxzen K.W., Knoll K.R., Wilson R.C., Strutt S.C., Kranzusch P.J. (2016). A bacterial Argonaute with noncanonical guide RNA specificity. Proc. Natl. Acad. Sci. U. S. A..

[156] Olovnikov I., Chan K., Sachidanandam R., Newman D.K., Aravin A.A. (2013). Bacterial argonaute samples the transcriptome to identify foreign DNA. Mol. Cell.

[157] Santovito D., Egea V., Bidzhekov K., Natarelli L., Mourao A., Blanchet X. (2020). Noncanonical inhibition of caspase-3 by a nuclear microRNA confers endothelial protection by autophagy in atherosclerosis. Sci. Transl. Med..

[158] Wang J.Y., Pausch P., Doudna J.A. (2022). Structural biology of CRISPR-Cas immunity and genome editing enzymes. Nat. Rev. Microbiol..

[159] Ding J., Lee Y.T., Bhandari Y., Schwieters C.D., Fan L., Yu P. (2023). Visualizing RNA conformational and architectural heterogeneity in solution. Nat. Commun..

[160] Tomezsko P.J., Corbin V.D.A., Gupta P., Swaminathan H., Glasgow M., Persad S. (2020). Determination of RNA structural diversity and its role in HIV-1 RNA splicing. Nature.

[161] Yang M., Zhu P., Cheema J., Bloomer R., Mikulski P., Liu Q. (2022). In vivo single-molecule analysis reveals COOLAIR RNA structural diversity. Nature.

[162] Ken M.L., Roy R., Geng A., Ganser L.R., Manghrani A., Cullen B.R. (2023). RNA conformational propensities determine cellular activity. Nature.

[163] Vicens Q., Kieft J.S. (2022). Thoughts on how to think (and talk) about RNA structure. Proc. Natl. Acad. Sci. U. S. A..

[164] Alfonzo J.D., Brown J.A., Byers P.H., Cheung V.G., Maraia R.J., Ross R.L. (2021). A call for direct sequencing of full-length RNAs to identify all modifications. Nat. Genet..

[165] Zhang S. (2021). MLC-Seq: de novo sequencing of full-length tRNAs and quantitative mapping of multiple RNA modifications. Res. Square.

[166] Lucas M.C., Pryszcz L.P., Medina R., Milenkovic I., Camacho N., Marchand V. (2023). Quantitative analysis of tRNA abundance and modifications by nanopore RNA sequencing. Nat. Biotechnol..

[167] Yamagami R., Sieg J.P., Assmann S.M., Bevilacqua P.C. (2022). Genome-wide analysis of the *in vivo* tRNA structurome reveals RNA structural and modification dynamics under heat stress. Proc. Natl. Acad. Sci. U. S. A..

[168] Butcher S.E., Pyle A.M. (2011). The molecular interactions that stabilize RNA tertiary structure: RNA motifs, patterns, and networks. Acc. Chem. Res..

[169] Roden C., Gladfelter A.S. (2021). RNA contributions to the form and function of biomolecular condensates. Nat. Rev. Mol. Cell Biol..

[170] Spitale R.C., Incarnato D. (2023). Probing the dynamic RNA structurome and its functions. Nat. Rev. Genet..

[171] Maraganore J. (2022). Reflections on Alnylam. Nat. Biotechnol..

[172] Bajan S., Hutvagner G. (2020). RNA-based therapeutics: from antisense oligonucleotides to miRNAs. Cells.

[173] Kilikevicius A., Meister G., Corey D.R. (2022). Reexamining assumptions about miRNA-guided gene silencing. Nucleic Acids Res..

[174] Childs-Disney J.L., Yang X., Gibaut Q.M.R., Tong Y., Batey R.T., Disney M.D. (2022). Targeting RNA structures with small molecules. Nat. Rev. Drug Discov..

[175] Jin H.Y., Gonzalez-Martin A., Miletic A.V., Lai M., Knight S., Sabouri-Ghomi M. (2015). Transfection of microRNA mimics should be used with caution. Front. Genet..

[176] Zhang Y., Shi J., Rassoulzadegan M., Tuorto F., Chen Q. (2019). Sperm RNA code programmes the metabolic health of offspring. Nat. Rev. Endocrinol..

[177] Reuter J.S., Mathews D.H. (2010). RNAstructure: software for RNA secondary structure prediction and analysis. BMC Bioinformatics.

[178] Biesiada M., Purzycka K.J., Szachniuk M., Blazewicz J., Adamiak R.W. (2016). Automated RNA 3D structure prediction with RNAComposer. Methods Mol. Biol..

[179] Kerpedjiev P., Hammer S., Hofacker I.L. (2015). Forna (force-directed RNA): simple and effective online RNA secondary structure diagrams. Bioinformatics.

[180] Pettersen E.F., Goddard T.D., Huang C.C., Couch G.S., Greenblatt D.M., Meng E.C. (2004). UCSF Chimera–a visualization system for exploratory research and analysis. J. Comput. Chem..

